# Conventional and pathogenic Th2 cells in inflammation, tissue repair, and fibrosis

**DOI:** 10.3389/fimmu.2022.945063

**Published:** 2022-08-09

**Authors:** Kota Kokubo, Atsushi Onodera, Masahiro Kiuchi, Kaori Tsuji, Kiyoshi Hirahara, Toshinori Nakayama

**Affiliations:** ^1^ Department of Immunology, Graduate School of Medicine, Chiba University, Chiba, Japan; ^2^ Institute for Advanced Academic Research, Chiba University, Chiba, Japan; ^3^ AMED-CREST, AMED, Chiba, Japan

**Keywords:** type 2 helper T (Th2) cell, interleukin-4 (IL-4), interleukin-5 (IL-5), interleukin-13 (IL-13), inflammation, tissue repair, tissue fibrosis, pathogenic Th2 (Tpath2) cell

## Abstract

Type 2 helper T (Th2) cells, a subset of CD4^+^ T cells, play an important role in the host defense against pathogens and allergens by producing Th2 cytokines, such as interleukin-4 (IL-4), IL-5, and IL-13, to trigger inflammatory responses. Emerging evidence reveals that Th2 cells also contribute to the repair of injured tissues after inflammatory reactions. However, when the tissue repair process becomes chronic, excessive, or uncontrolled, pathological fibrosis is induced, leading to organ failure and death. Thus, proper control of Th2 cells is needed for complete tissue repair without the induction of fibrosis. Recently, the existence of pathogenic Th2 (Tpath2) cells has been revealed. Tpath2 cells produce large amounts of Th2 cytokines and induce type 2 inflammation when activated by antigen exposure or tissue injury. In recent studies, Tpath2 cells are suggested to play a central role in the induction of type 2 inflammation whereas the role of Tpath2 cells in tissue repair and fibrosis has been less reported in comparison to conventional Th2 cells. In this review, we discuss the roles of conventional Th2 cells and pathogenic Th2 cells in the sequence of tissue inflammation, repair, and fibrosis.

## Introduction

Type 2 helper T (Th2) cells are a subset of CD4^+^ T cells characterized by the production of Th2 cytokines, including interleukin-4 (IL-4), IL-5, and IL-13 (1). The Th1-Th2 paradigm was first described about 30 years ago (2), and for a long time before that, type 2 immunity was thought to be a simple counter-regulatory mechanism that antagonizes type 1 immunity (3). Now, however, Th2 cells are often observed in tissues in allergic patients and are known to play critical roles in the pathogenesis of allergic diseases (1, 4).

It was recently shown that Th2 cells include pathogenic Th2 (Tpath2) cells that highly express the receptor for IL-33 (a cytokine that is released during tissue injury) and produce large amounts of IL-5 (1). Thus, the “pathogenic Th population disease-inducing model” has been proposed, in which Tpath2 cells are important promoters of allergic inflammation in both mouse and human systems (1, 5). Th2 cells are also involved in the repair of tissues that are damaged by parasitic infections, house dust mite (HDM), and air pollution. IL-4 and IL-13, which are induced by Th2 cells, activate macrophages and epithelial cells and enhance the production of extracellular matrix (ECM), an element crucial for tissue repair (6–10). However, when the tissue repair process becomes chronic, excessive, or uncontrolled, it may induce the development of pathological fibrosis in various organ systems.

The mechanisms underlying the induction of fibrosis by Th2 cells have been reported to include deposition of ECM by fibroblasts in response to various cytokines and growth factors, including Th2 cytokines (11), but the detailed cellular and molecular mechanisms remain unclear. Currently, research to elucidate the mechanism underlying Th2 cell-mediated fibrosis is being actively conducted. Recently, we have reported that eosinophil reprogramming *via* IL-5 and amphiregulin production by Tpath2 cells (12) and a CD103^low^ cell population among tissue-resident CD4^+^ T cells (13) were also found to promote tissue fibrosis.

We herein review the role of Th2 cells, including the conventional, pathogenic, and tissue-resident Th2 cells that we identified, in the sequence of tissue inflammation, repair, and fibrosis, and discuss how tissue fibrosis can be controlled.

## Th2 cells in the induction of inflammation

### Induction of Th2 cell differentiation

Th2 cells produce so-called Th2 cytokines: IL-4, IL-5, and IL-13. These cytokines play an important role in humoral immunity and helminthic infection defense but are also central to the pathogenesis of many allergic inflammatory diseases.

Peripheral naïve CD4^+^ T cells, upon recognition of antigenic peptides by the T cell receptor (TCR), migrate into lymphoid tissues and functionally differentiate into different Th cell subsets. When CD4^+^ T cells receive IL-4 that is released by innate immune cells, such as group 2 innate immune system lymphocytes (ILC2s), mast cells, and basophils in lymphoid tissues, these CD4^+^ T cells differentiate into Th2 cells through IL-4-induced phosphorylation of STAT6 and the increased expression of GATA3 (14–19). GATA3 protein stability is also important for the functions of Th2 cells (20, 21). Through direct or indirect interaction with epigenetic modifiers, GATA3 is involved in chromatin remodeling at the Th2 cytokine gene loci: histone H3K9 acetylation (22, 23), histone H3K4 methylation (20), and DNA demethylation (24). In addition, epigenetic modifications of the *Gata3* gene control the stable expression of GATA3 proteins (20, 25, 26). The TCR-mediated signaling pathway also contributes to Th2 cell differentiation (27). T cells that lack nuclear factor of activated T-cells 2 (NFAT2), encoded by the *Nfatc1* gene, produce less IL-4, indicating that the TCR-dependent activation of NFAT2 plays a role in Th2 cell differentiation (28). Prolonged TCR signaling is proposed to enhance the pathogenicity of Th2 cells due to a lack of *Klf2* gene reactivation (29, 30).

**Figure 1:**
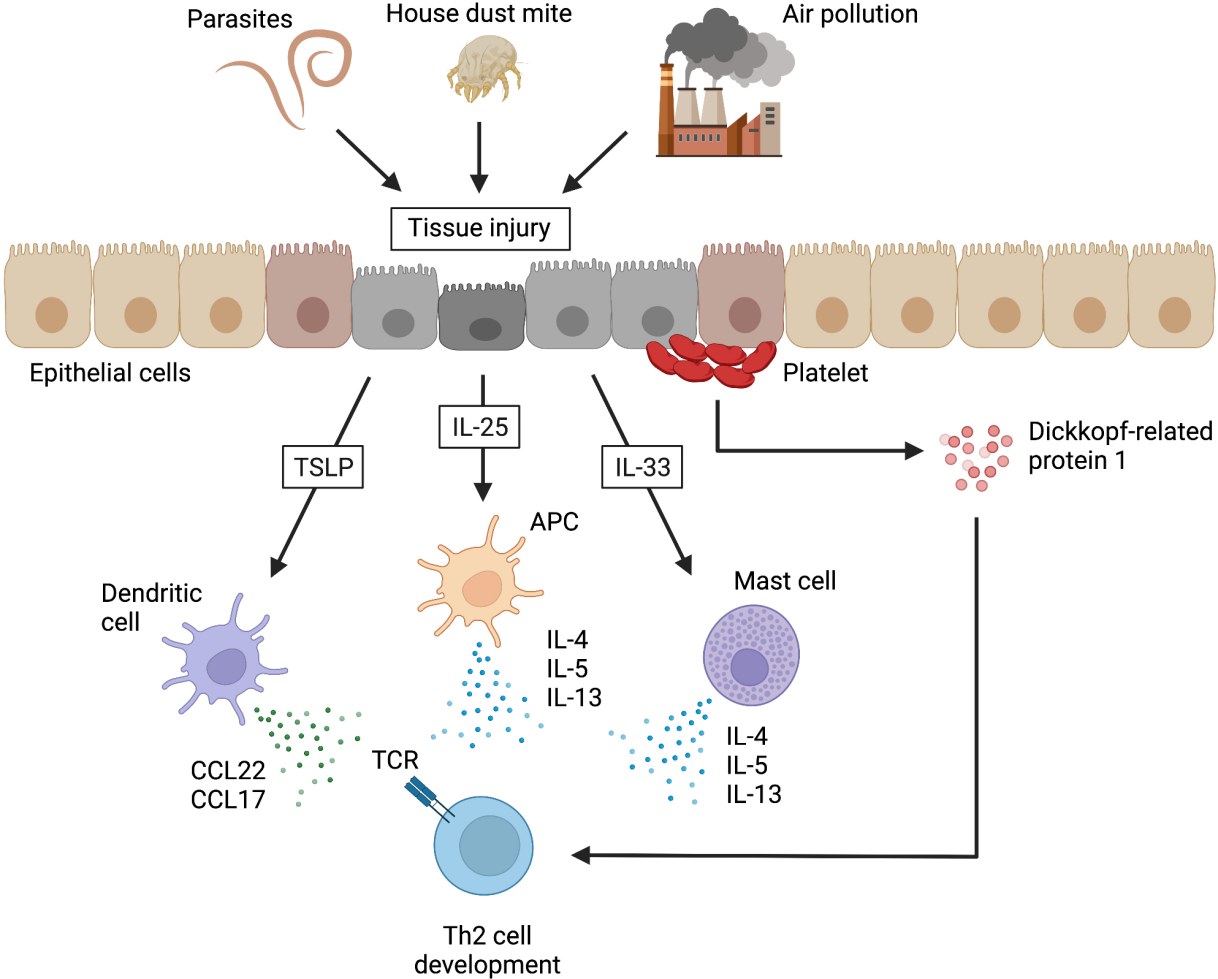
Alarmins that induce Th2 cell development in non-lymphoid tissues. When airway epithelial tissues are damaged by helminth infections, house dust mites, or air pollution, the damaged epithelial cells release alarmins (e.g., thymic stromal lymphoid neoplastic factor [TSLP], interleukin [IL]-25, and IL-33). TSLP activates local dendritic cells to secrete CC chemokine ligand 22 (CCL22) and CCL17, which promote the development of type 2 helper T (Th2) cells. IL-25 induces the production of IL-4, IL-5, and IL-13 from antigen-presenting cells (APCs), resulting in the promotion of Th2 cell development. IL-33 also promotes Th2 cell development by inducing the production of Th2 cytokines from mast cells. In addition, Dickkopf-related protein 1, which is a Wnt antagonist released from platelets at the site of injury, can similarly promote Th2 cell development. TCR, T cell receptor. This figure was created with BioRender.com.

When airway epithelial tissues are damaged by helminth infection, HDM, or air pollution, the innate immune system is activated by the release of alarmin cytokines, such as thymic stromal lymphoid neoplastic factor (TSLP), IL-25, and IL-33 from the damaged epithelial cells. In addition to IL-4, these alarmin cytokines have been reported to be important for inducing Th2 responses under various conditions *in vivo* and *in vitro* (31). TSLP activates local dendritic cells to secrete CC chemokine ligand 22 (CCL22) and CCL17, which promotes Th2 cell differentiation. IL-25 induces the production of IL-4, IL-5, and IL-13 from antigen-presenting cells and promotes the differentiation of Th2 cells (31). In addition, the production of IL-5 from CD4^+^ T cells was enhanced in polyps of eosinophilic chronic rhinosinusitis (ECRS) patients by direct stimulation by increased IL-25, as Tpath2 cells also express the receptor for IL-25 (32). IL-33 activates NF-κB and MAP kinase *via* the IL-1 receptor-like 1 (ST2) and induces Th2 cytokine production in mast cells (33). Recent studies have shown that in addition to IL-4, the Wnt antagonist, platelet-derived Dickkopf-related protein 1 (DKK1)—in combination with TCR involvement—is an important contributor to Th2 cell differentiation in pathological type 2 cell-mediated inflammation (34). In addition to the well-known fact that platelets are an important source of platelet-derived growth factor (PDGF), there are mechanistic and physiological links among local tissue damage, platelet activation, and Th2 cell differentiation (**Figure 1**). Finally, the gene expression pattern as a Th2 subset that is acquired by the above-described mechanism is maintained by epigenetic modifications (25, 35).

### Inflammatory response induced by conventional Th2 cells

**Figure 2:**
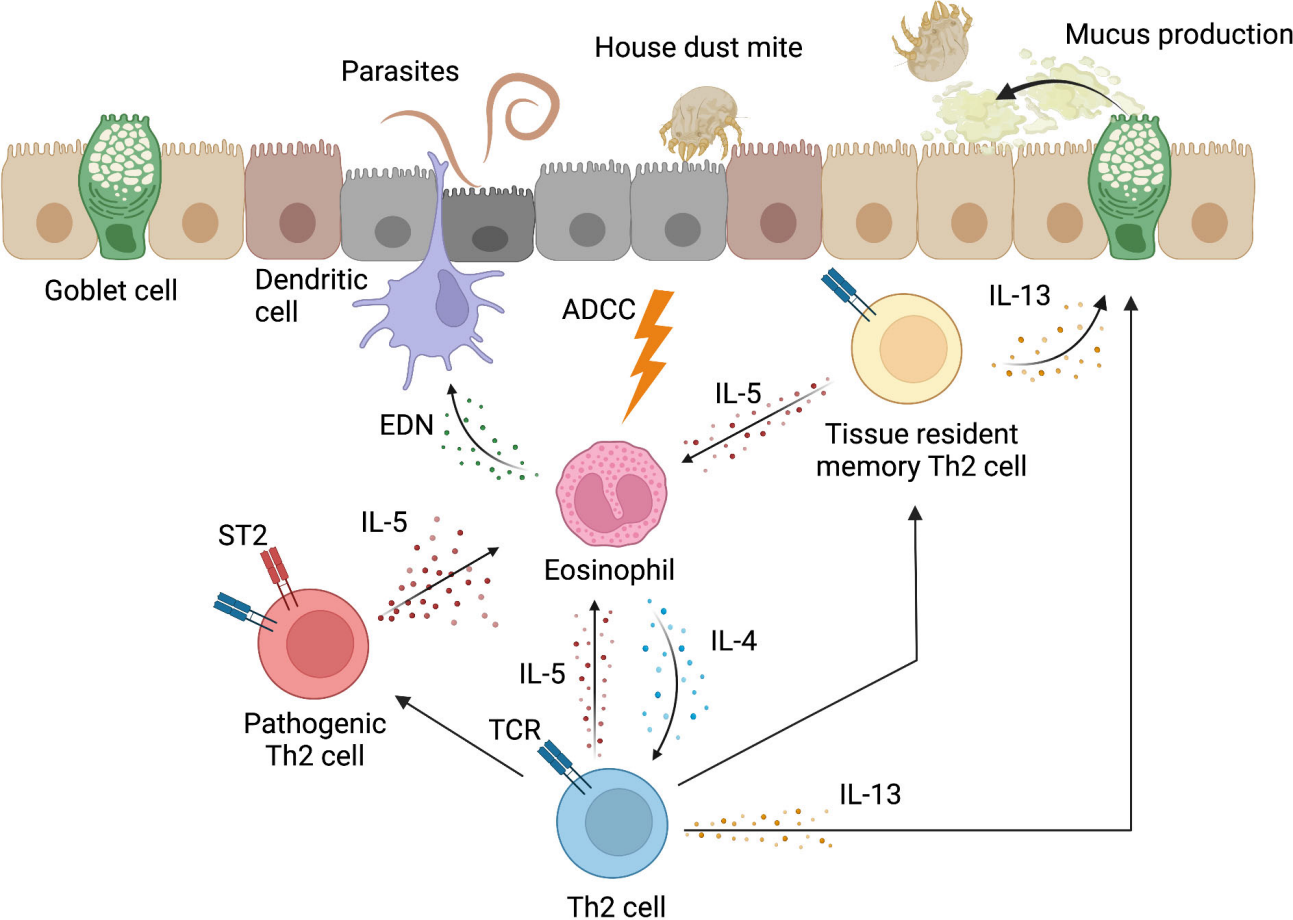
Inflammatory response induced by Th2 cells. In inflammatory tissues, Th2 cells induce an inflammatory response to eliminate antigens. Th2 cells recruit eosinophils to the site of inflammation through IL-5 production and promote their survival. Eosinophil-derived neurotoxin (EDN) acts as a chemoattractant and activator of dendritic cells. Eosinophils can also kill some parasites *via* the classical antibody-dependent cytotoxicity (ADCC) mechanism. In addition, eosinophils promote the development of Th2 cells through the production of IL-4. In addition to the induction of eosinophilic inflammation by IL-5, Th2 cells produce IL-13, which induces mucus production from epithelial cells, including goblet cells, and promotes the elimination of allergens from epithelial tissues. The Th2 cell population contains pathogenic Th2 (Tpath2) cells that highly express ST2, which is the receptor for IL-33. Tpath2 cells become high-producers of IL-5 when activated by IL-33. Tissue-resident memory Th2 cells are differentiated from a portion of Th2 cells, and play an important role in type 2 inflammatory responses in local tissues through the production of Th2 cytokines, such as IL-5 and IL-13. This figure was created with BioRender.com.

Effector Th2 cells are primed in lymphoid tissues and eventually migrate into inflammatory tissues (36). CD69, a type II transmembrane glycoprotein with C-type lectin domains expressed on activated CD4^+^ T cells, is important for migration into inflammatory tissues (37). CD69 helps CD4^+^ T cells localize to inflammatory tissues by binding to a functional ligand for CD69, Myosin Light Chain 9 (Myl9), which is contained in the intravascular net-like structures that are formed in inflammatory tissues (38, 39). Th2 cells that migrate to inflammatory tissues are able to exert an effector function when exposed to factors in their environment. In particular, in the absence of signals from epithelial-derived cytokines (TSLP, IL25, and IL33), Th2 cells in the lung do not express IL-5 and IL-13, and are unable to exert full effector functions (40). Th2 cells that have an acquired effector function can produce Th2 cytokines, including IL-4, IL-13, and IL-5. IL-4 induces Th2 cell differentiation in an autocrine manner at inflammatory sites (41). IL-13 promotes allergen elimination from the epithelium by inducing the accumulation of mucin in the epithelium (42, 43). IL-5 recruits eosinophils to inflammatory sites and also promotes their survival (44). Eosinophil-derived neurotoxin (EDN) in granule proteins acts as an activator and chemoattractant of dendritic cells, and consequently, activated dendritic cells enhance Th2 responses. In addition, eosinophils can kill some parasites *via* the classical antibody-dependent cytotoxicity (ADCC) mechanism (44, 45). Furthermore, eosinophils are sources of Th2-inducing cytokines (mainly IL-4 and IL-13) in the very early stage of the immune response in lymph nodes and tissues and can induce Th2 differentiation by antigen presentation to CD4^+^ T cells (46–48) (**Figure 2**). ILC2s are also associated with the induction of type 2 inflammation by directly producing Th2 cytokines and activating Th2 cells *via* MHCII and IL-13 (49). ILC2s produce large amounts of Th2 cytokines upon stimulation by IL-25, IL-33 and TSLP, and also constitutively produce a certain amount of IL-5 (50, 51).

### Identification of pathogenic Th2 (Tpath2) cells

One of the major advances in the understanding of the induction of tissue inflammation by Th2 cells is the identification of Tpath2 cells (1, 52–57). Tpath2 cells express high levels of ST2, a component of the IL-33 receptor, and when activated by IL-33 released from damaged epithelial cells, they become a high producer of IL-5 (56). IL-33 is proposed to be required for optimal human pathogenic Th2 cell effector function (57). The IL-5 produced by Tpath2 cells exacerbates the pathology of eosinophilic inflammation in both mouse and humans (53). In fact, mepolizumab and benralizumab, which are humanized monoclonal antibodies targeting IL-5 and IL-5Rα, respectively, have both shown remarkable therapeutic effects in various types of eosinophilic diseases, including ECRS and asthma (58). In addition to IL-33, IL-7 maintains memory Tpath2 cells in an ectopic lymphoid tissue called “inducible bronchus-associated lymphoid tissue (iBALT)” in murine inflamed lung tissue (59). Recent technical advances, including the genome-wide analysis of single-cell transcriptomes, regulomes and proteomes, have highlighted the diversity and detailed characteristics of Tpath2 cells (60, 61). We herein summarize the current understanding of pathogenic Th2 cell markers other than ST2 and IL-5 (62).

A CC chemokine, CCL8, is highly expressed in the skin and functions as a ligand for the chemokine receptor, CCR8. CCR8^+^ memory-type Th2 cells have been identified as key pathogenic players in chronic skin inflammation in a mouse model of skin inflammation. In addition, a similar cell type that produces higher levels of IL-5 is found in healthy human donors, suggesting that CCR8 could be a marker for pathogenic Th2 cells (52). CRTH2 is a G-protein coupled receptor with which Tpath2 cells sense and react to prostaglandin D2, a pleiotropic mediator for activation, migration, and cytokine production in type 2 immune responses (62). Importantly, human pathogenic Th2 cells also express hematopoietic prostaglandin D synthase (HPGDS), which catalyzes the conversion of prostaglandin H2 to D2. Thus, Tpath2 cells are able to amplify their own activation and effector function *via* the HPGDS-PGD2-CRTH2 axis (53, 63). CD161 is a C-type lectin-like receptor and plays an inhibitory role on NK cell cytotoxicity. The ligand of CD161 is the C-type lectin domain family 2 member D (CLEC2D or LLT1), which is expressed in human epithelial cell lines isolated from the lung, suggesting that pathogenic Th2 cells might be activated *via* CD161 and contribute to inflammation in the lung (64). The receptor of IL-25, which consists of IL-17 receptor B (IL-17RB) and IL-17RA, also serves as a marker of the human pathogenic Th2 cells, since IL-25 stimulates Tpath2 proliferation and cytokine production (54).

Lipid metabolism-related molecules are also involved in the induction of pathogenic Th2 cells. For example, peroxisome proliferator-activated receptor gamma (PPAR-γ) is a fatty acid-activated nuclear receptor known for controlling adipogenesis, lipid and glucose metabolism, and inflammation. The *PPARG* gene is reproducibly reported as a marker associated with the human pathogenic Th2 phenotype (55, 65–67), while specific endogenous PPAR-γ ligands remain to be identified. In humans, the roles of PPAR-γ in pathogenic Th2 cells remain unclear. However, the specific functional role of PPAR-γ in pathogenic Th2 cells has been characterized in mice: the upregulation of IL-33 receptor ST2 and the activation of Tpath2 cells in allergic inflammation (68). Indeed, mice lacking PPAR-γ in T cells showed impaired immune responses in both allergic lung inflammation and parasite infection (69). Another example of the markers associated with lipid metabolism is free fatty acid receptor 3 (FFAR3), which encodes a G protein-coupled receptor for short chain fatty acids. Multiple studies have shown that human pathogenic Th2 cells express FFAR3, the expression levels of which were correlated with IL-5 levels and the number of tissue eosinophils (55, 65, 66).

### Tpath2 cells in inflammatory diseases

As described above, the concept of Tpath2 cells has been recognized as fundamental for understanding the pathogenesis of inflammatory diseases, including allergic inflammation of the upper and lower airways, atopic dermatitis, and gastrointestinal inflammation in humans (39, 70).

While conventional Th2 cells have long been implicated in allergic airway inflammation, recent studies in human patients indicate that the disease is specifically driven by pathogenic Th2 cells (63, 66, 67). This idea is further supported by the successful clinical application of anti-IL-5 monoclonal antibodies in the treatment of asthma patients (56). In addition, other therapeutics targeting alarmins that activate pathogenic Th2 cells, such as anti-TSLP and anti-IL-33 treatments, are being developed for allergic asthma (71). Moreover, inhibitors of CRTH2 are currently being evaluated in clinical trials for allergic asthma (62). In ECRS patients, IL-5-producing pathogenic Th2 cells have also been identified in the upper airways (32).

Evidence that the pathogenic Th2 cell subset might play a role in atopic dermatitis (AD) came from a mouse model in which chronic skin inflammation was found to be driven by a specific subset of CCR8^+^ memory Th2 cells expressing high levels of IL-5 (52). Consistent with these findings, CCL18, a ligand of CCR8, is shown to be overexpressed in the skin of AD patients and to be well-correlated with disease activity (72). However, treatment with a monoclonal antibody to human interleukin-5 (mepolizumab) did not result in clinical success in patients with AD (73), whereas blocking of the Th2 cytokine signaling with a monoclonal antibody against the IL-4/IL-13 receptor alpha chain shows high efficacy in patients with severe AD (74).

Recently, a single cell analysis of T cells isolated from eosinophilic esophagitis (EoE) tissue in humans identified a pathogenic Th2 cell population that expresses HPGDS, CRTH2, and CD161. These pathogenic Th2 cells produce higher levels of IL-5 and IL-13, which are correlated with disease severity (65). Thus, EoE has been considered as one of the diseases associated with pathogenic Th2 cells. In another study, CRTH2^hi^CD161^hi^HPGDS^hi^ST2^+^ Tpath2 cells were proposed to be a pathogenic T-cell population in EoE patients (53). Furthermore, the effects of allergen-specific immunotherapy in EoE patients have revealed a correlation between the presence of CD45RO^hi^CRTH2^hi^CCD49d^hi^CCR4^hi^CXCR3^lo^CCR6^lo^CD27^lo^ST2^+^ Tpath2 cells and disease activity (55). G-protein-coupled receptor 15 (GPR15) is a known lymphocyte trafficking receptor and may serve as a pathogenic marker in inflammatory bowel disease: increased numbers of CD4^+^CD45RO^+^GPR15^+^ memory-type Th2 cells are found in the colon of patients with ulcerative colitis (75, 76).

### Tpath2 and tissue-resident memory Th2 cells

Memory Th2 cells, which are differentiated from effector Th2 cells, are central to adaptive immunity against parasite infection and play an important role in the host defense through inflammatory responses (36). Memory Th2 cells have also been implicated in the pathogenesis of chronic inflammatory diseases (1). Evidence from recent studies indicates that memory Th2 cells, including pathogenic memory Th2 cells, are more heterogeneous in their function than previously recognized. Thus, a precise understanding of the nature of the involvement of memory Th cells in various chronic inflammatory diseases is quite important for the development of new therapeutic approaches for intractable immune-related disorders. More recently, it has become clear that there are two types of memory CD4^+^ T cells: circulating and non-circulating (77). The non-circulating cells are now called tissue-resident memory T (T_RM_) cells (78, 79). Unlike circulating memory T cells, which circulate throughout the body *via* blood vessels and lymphatic vessels, T_RM_ cells are retained in non-lymphoid tissues, such as the lungs, intestines, and skin (78). In the lungs of mice in which inflammation is induced by HDM, Th2 T_RM_ cells are transcriptionally and functionally distinct from circulating memory Th2 cells (80). Th2 T_RM_ cells have increased expression of *Il5* and *Il13* (80). In addition, in the lungs of mice, CD4^+^ T_RM_ cells that were induced by repeated exposure to *Aspergillus fumigatus* caused an inflammatory response (13). Thus, Th2 T_RM_ cells play an important role in type 2 inflammatory responses in local tissues (81) (**Figure 2**).

In the skin, T_RM_ provide frontline immune defense (82, 83). Recently, a transcriptome analysis of human CD4^+^ T_RM_ from healthy skin revealed an interesting overlap of the T_RM_ transcriptome with that of pathogenic Th2 cells (84). For example, human CD4^+^ T_RM_ were found to express specific molecules, including markers described in the previous section—CCR8 and PPAR-γ—which are closely linked to pathogenic Th2 cells. The biological relevance of this remarkable similarity remains to be elucidated; however, the result indicates that both T_RM_ and pathogenic Th2 cells share a similar differentiation program for adaptation to the tissue environment of the skin. Interestingly, in mice, PPAR-γ mediates metabolic adaptation of CD8^+^ T_RM_ to the lipid-rich microenvironment of the skin (23). Since both T_RM_ and pathogenic Th2 cells are linked to the chronic inflammation of barrier tissues, the similarity in the transcriptome could be a hint to future therapeutic or even curative strategies.

## Th2 cells in the tissue repair

**Figure 3:**
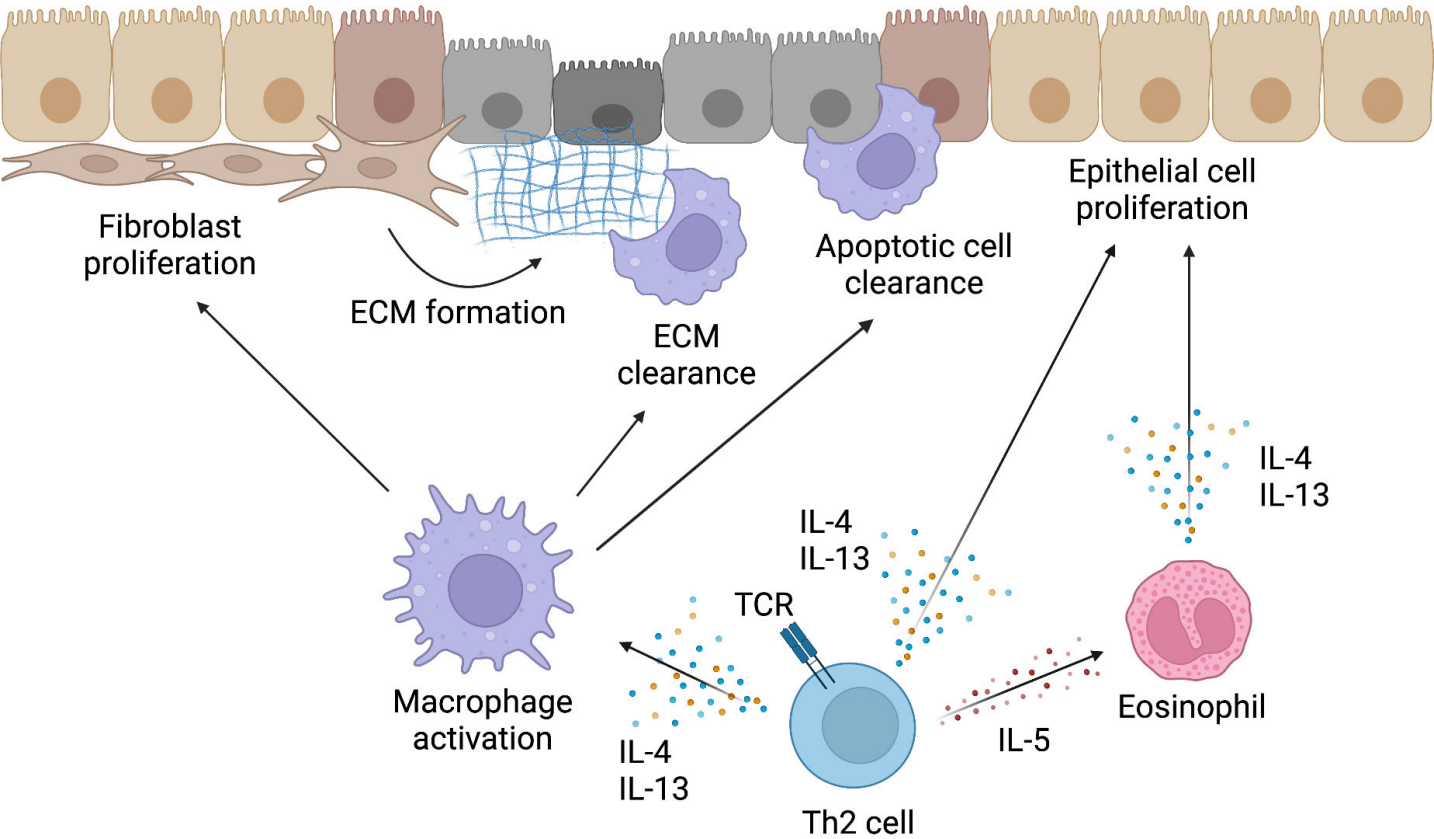
Mechanism of tissue repair by Th2 cells. Type 2 cytokines produced by Th2 cells not only induce type 2 inflammatory responses but also contribute to the repair of injured tissues. The activation of macrophages by IL-4 and IL-13 is important for the resolution of the type 2 inflammatory response and initiation of tissue repair. Activated macrophages induce fibroblast proliferation, remove extracellular matrix (ECM) formed by fibroblasts and other cells, and clear apoptotic cells in injured tissues. IL-4 and IL-13 produced by Th2 cells can also directly induce the proliferation of epithelial cells and contribute to the reconstruction of injured tissues. Furthermore, eosinophils, which are recruited to inflammatory sites by IL-5 produced by Th2 cells, can also produce IL-4 and IL-13. Thus, Th2 cells induce tissue repair both directly and indirectly. This figure was created with BioRender.com.

In addition to inducing type 2 inflammatory responses, type 2 cytokines that are produced by Th2 cells also directly or indirectly help the repair of injured tissues by targeting a wide variety of immune and non-immune cells, including fibroblasts, epithelial cells, macrophages, and endothelial cells (**Figure 3**). Tissue repair is a tightly coordinated and highly dynamic process that restores tissues injured by parasitic infections, HDM, air pollution, and other conditions to their original state. We herein summarize the outline of the current understanding of the tissue repair processes, which are thought to be mainly conventional Th2 cells.

Basically, this process can be divided into three distinct but overlapping phases. These three stages are defined as “coagulation and inflammation”, “tissue formation”, and “tissue reconstruction” (85). In the “coagulation and inflammation” phase, the injury site is first isolated from outside the body by blood coagulation, and then the mobilization of inflammatory cells, such as Th2 cells, begins (85). The activation of macrophages by type 2 cytokines, such as IL-4 and IL-13, is important in the resolution of the subsequent stages of inflammation and in tissue formation and remodeling (6, 7).

In the second stage, “tissue formation”, pro-inflammatory signals, such as type 2 cytokines, weaken, and cell proliferation is initiated by growth factors, such as transforming growth factor-β (TGF-β) and basic fibroblast growth factor (FGF-2). At this stage, two processes are thought to be important: the removal of apoptotic cell debris by activated macrophages and the induction of fibroblast proliferation (86). In fact, wound healing is impaired when the number of macrophages is reduced (87). In particular, macrophages that receive type 2 cytokines (also referred as to an alternatively activated or M2 macrophages) are involved in tissue repair in the liver, central nervous system (CNS), heart, skeletal muscle, and lung (88). In mice, IL-4 produced by basophils and Th2 cells activates type 2 immune responses that convert the recruited inflammatory monocytes into wound-healing macrophages in the liver (89). These macrophages can replace the lost Kupffer cells in the liver and restore tissue homeostasis (89). Furthermore, the IL-4-activated macrophages are highly metabolically active and quickly consume critical amino acids in the injury site, which can slow the progression of fibrosis. This is due to the suppression of local CD4^+^ T cell proliferation and myofibroblast activation (90, 91). In the CNS of mice, depletion of intralesional IL-4-associated macrophages substantially delayed oligodendrocyte differentiation (92). In addition, multipotent adult progenitor cells protect against axonal dieback in spinal cord injury by expanding protective IL-4-associated macrophages in rats (93). In the heart, the critical contribution of IL-4- and IL-13-activated macrophages to cardiac repair was reported (94): Trib1-deficient mice that selectively lack IL-4-associated macrophages frequently experienced cardiac rupture after myocardial infarction. In the skeletal muscle in a mouse model of Duchenne muscular dystrophy, which is associated with IFN-γ-activated macrophages, IFN-γ-deficiency markedly improved the motor function in the late regenerative phase. It was associated with a skewing of macrophages toward an IL-4-associated anti-inflammatory phenotype (95). In humans, myogenic precursor cell activation by anti-inflammatory macrophages is proposed to be crucial to muscle cell regeneration following injury (96). Taken together, these results suggest a possible protective role of reparative macrophages in the skeletal muscle.

Eosinophils are also involved in tissue repair. For example, eosinophils induce hepatocyte proliferation by secreting IL-4 and IL-13 in an IL-4Rα-dependent manner during the tissue repair process in the liver (97). IL-4 and IL-13 also induce the proliferation of epithelial cells that express IL-4Rα, which promotes stem and progenitor cells in adult tissues to proliferate and differentiate (8, 9). Similar findings have also been reported in cases of skeletal muscle injury in a mouse model. In skeletal muscle injury, IL-4, which is derived from eosinophils, targets the regenerative function of muscle-resident adipocyte progenitors and fibroblasts that contribute to myogenesis (98). As described in **Figure 2**, IL-5, which is essential for the eosinophil function, is produced in large amounts by Tpath2 cells. Since Tpath2 cells are activated by IL-33 released from injured tissues in both mice and humans (39), the IL-33-Tpath2 axis is expected to play an important role in the repair of injured tissues by collaborating with macrophages and eosinophils. For example, IL-33 was shown to attenuate experimental autoimmune encephalomyelitis (EAE) in mice by suppressing IL-17 and IFN-γ production and by inducing reparative IL-4-associated macrophages, suggesting that type 2 cytokines produced by Tpath2 cells polarize anti-inflammatory macrophages (99). As targets of IL-33, ILC2s also contribute to tissue repair. In allergic (100–102) and infection models (40, 103), ILC2s, in addition to Th2 cells, have been reported to be a source of IL-13. In particular, ILC2s produce large amounts of IL-13 when stimulated by alarmin released during tissue injury (50). Lung ILC2s also produce amphiregulin in response to IL-33 and restore the respiratory function after influenza virus-induced lung injury (104).

In addition to the dynamic proliferation and differentiation of these cells, the formation of *de novo* ECM and the deposition of collagen supporting the migrated cells are also hallmarks of the “tissue formation” phase. In tissue repair, the ECM, which includes components such as collagen and fibronectin, is important because it mechanically stabilizes the damaged tissue, anchors growth factors, and serves as a scaffold for endothelial cells, immune cells, and fibroblasts and to migrate to the site of tissue damage or repair (10). The ECM can also bind growth factors, thus acting as a reservoir for growth factors, concentrating their activity close to the cell and protecting it from degradation (105). Through the action of tissue inhibitor of metalloproteinases (TIMPs) and matrix metalloproteinases (MMPs), myofibroblasts continuously regulate matrix deposition and turnover (106, 107). This process is organized by cell-to-cell and cell-to-ECM interactions.

The third stage, “tissue reconstruction,” is the final stage of tissue repair. In this stage, the tissue is reconstructed to its original form by the wound reconstruction process. Wound tissue reconstruction is a multi-step process. During this stage, which can last from several weeks to several months, myofibroblasts, macrophages, and endothelial cells present in the tissue undergo apoptosis, and newly formed blood vessels regress (85, 108). The newly formed collagen layer beneath the wound undergoes remodeling and reconstruction. During this phase, macrophages contribute to tissue repair by ingesting cellular debris and decomposing the excess ECM that has accumulated in and around the wound (108). Nonetheless, the depletion of skin macrophages during the tissue reconstruction phase does not significantly affect the outcome of tissue repair. This suggests that macrophages have redundant roles in tissue repair and that their function may overlap with the functions of other cell populations at this wound repair phase (109). However, how tissue repair is regulated by macrophages that are activated by type 2 cytokines and other cell types that characterize type 2 inflammation, such as mast cells, basophils, eosinophils, Th2 cells, and ILC2s, remains unclear.

## Th2 cells in the induction of fibrosis

### Overview of tissue fibrosis

Fibrosis is not a disease, rather it is the result of uncontrolled tissue repair responses that occur after many types of tissue injury, particularly caused by chronic inflammatory disease. Fibrosis is the widespread accumulation of fibrous connective tissue and is defined by the excessive accumulation of ECM components, such as collagen and fibronectin (110). In fact, the formation of fibrotic tissue is a normal and critical step in tissue repair in all organs in response to tissue injury caused by various triggers, such as infection, inflammation, autoimmune diseases, degenerative diseases, and tumors (111). When tissue is injured, the wound healing response is initiated through mechanisms that were described previously. If the injury is mild or non-repetitive, the deposition of ECM components increases only temporarily, allowing for efficient wound healing and facilitating the functional recovery of tissue structures (106, 107). However, if the damage is repeated or severe, wound healing mechanisms may become uncontrollable and ECM components may continue to accumulate, resulting in the destruction of tissue structures, organ dysfunction, and eventually organ failure (110, 112). In various diseases, such as hepatic cirrhosis, renal interstitial fibrosis, pulmonary fibrosis, myocardial infarction, graft-versus-host disease (GVHD), and systemic sclerosis, fibrotic remodeling impairs the organ function and causes high morbidity and mortality (111). Although the role of macrophages in fibrogenesis has been studied for many years, the mechanisms through which T cells regulate fibrogenesis are not fully understood.

### Mechanism underlying lung fibrosis induced by Tpath2 cells

**Figure 4:**
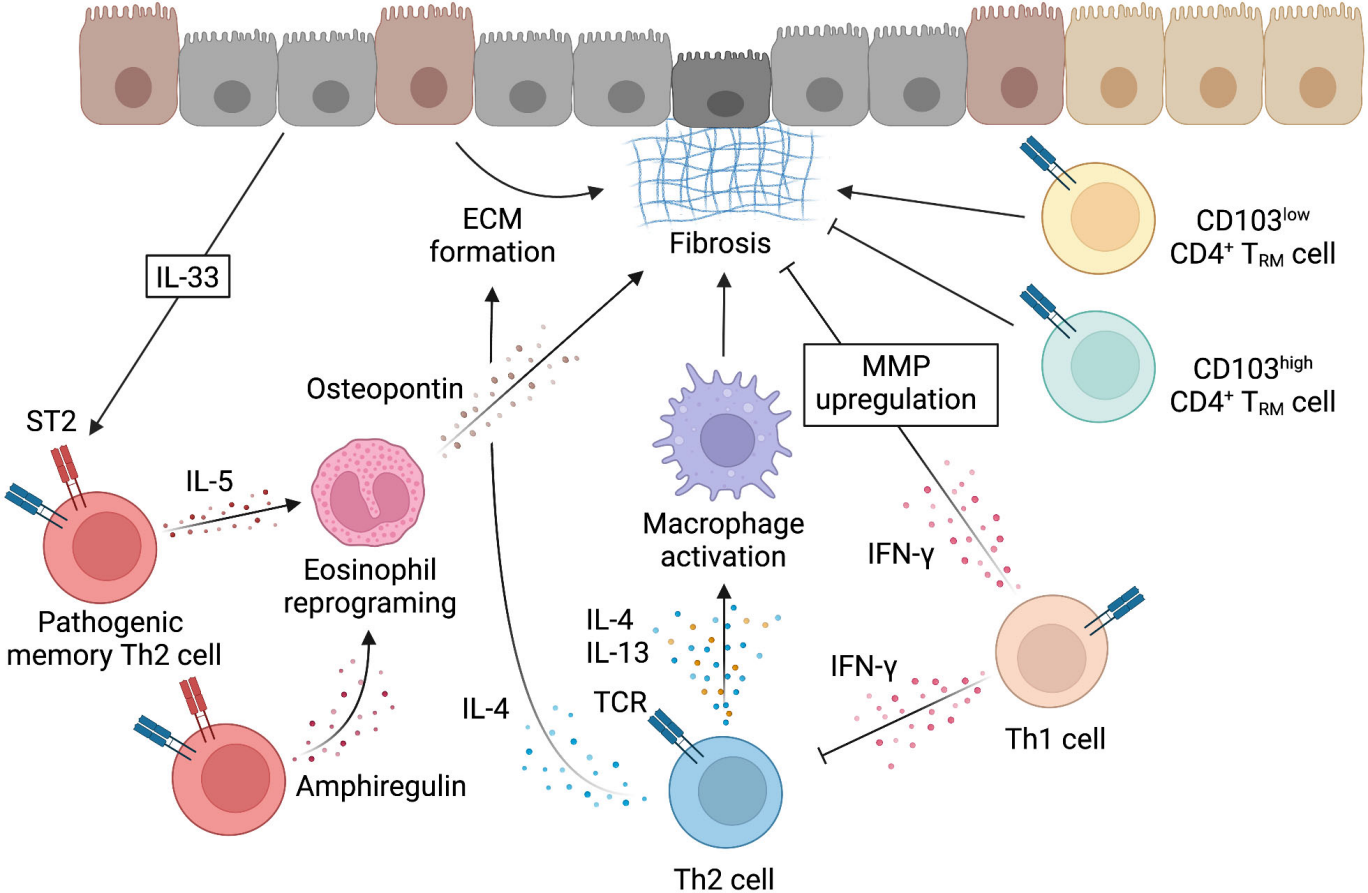
Mechanism underlying the induction of fibrosis by Th2 cells. Fibrosis can be induced when the tissue repair response by cells such as Th2 cells becomes uncontrollable. While macrophages that are activated by IL-4 and IL-13 work for tissue repair through the mechanisms described in Figure 3, fibrosis is induced by various mechanisms. IL-4 can also directly induce fibrosis by acting on epithelial cells. IL-33, which is produced by injured epithelial cells, activates memory-type pathogenic Th2 cells *via* ST2. Memory-type pathogenic Th2 cells include a subpopulation that produces IL-5 and a subpopulation that produces amphiregulin in response to IL-33 stimulation. Amphiregulin acts on eosinophils recruited by IL-5 and reprograms eosinophils to produce osteopontin and induce fibrosis. CD4^+^ T cells that are involved in fibrosis also include tissue-resident memory T (T_RM_) cells with the low or high expression of CD103. CD4^+^ T_RM_ cells with the low expression of CD103 produce high levels of Th2 cytokines and promote fibrosis, whereas CD4^+^ T_RM_ cells with the high expression of CD103 express high levels of Foxp3 and inhibit fibrosis. IFN-γ produced by Th1 cells has been reported to inhibit the induction of fibrosis by Th2 cells. IFN-γ may directly inhibit fibrosis by upregulating the expression of matrix metalloproteinases (MMP) that degrade ECM. This figure was created with BioRender.com.

**Figure 5:**
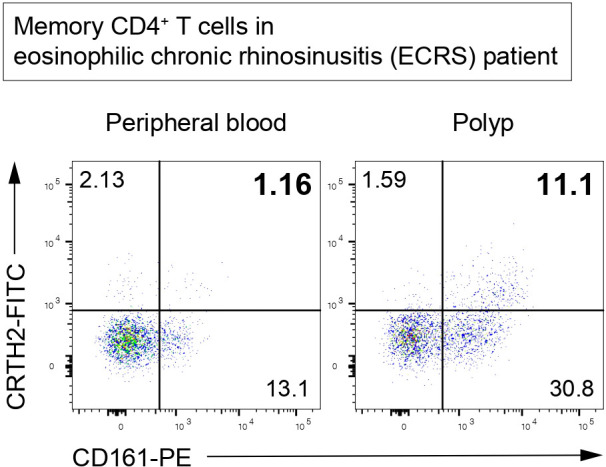
Memory-type Tpath2 cells in an ECRS patient. Representative plots of CD4^+^CD45RO^+^ (memory CD4^+^) T cells isolated from the peripheral blood and polyp of an ECRS patient. CRTH2-positive and CD161-positive cells are defined as Tpath2 cells (12).

Memory-type Tpath2 cells express high levels of the IL-33 receptor ST2 and play important roles in fibrogenesis in both HDM-induced murine lung inflammation and human airway inflammatory disease (5, 12) (**Figure 4**). In mouse, IL-33, which is mainly produced by epithelial cells undergoing tissue injury, activates memory-type Tpath2 cells and induces the production of amphiregulin, a member of the epidermal growth factor family (12). In HDM-induced murine lung inflammation, amphiregulin reprograms the eosinophil transcriptome *via* the epidermal growth factor receptor (EGFR) and promotes the production of osteopontin, which is a fibrosis-inducing protein (12, 113). Amphiregulin-producing memory-type Tpath2 cells and IL-5-producing memory-type Tpath2 cells were thought to be distinct subpopulations and to cooperate to induce eosinophilic inflammation and to establish airway fibrosis (12). In addition, an analysis of nasal polyps from patients with ECRS showed that the percentages of memory-type Tpath2 cells in nasal polyps were higher than those in peripheral blood (**Figure 5**). Furthermore, in nasal polyps of ECRS patients, amphiregulin-producing memory CD4^+^ T cells and osteopontin-producing eosinophils were found, with a dense fibrotic response (12, 114). Pharmacological blocking of EGFR signaling attenuates the expression of *Spp1*, encoding osteopontin, in both mouse and human eosinophils (12). Thus, the IL-33-amphiregulin-osteopontin axis induces a fibrotic response in chronic allergic inflammation in both mouse and human systems (12). In contrast, amphiregulin that is produced from activated lung ILC2s restores the respiratory function after influenza virus-induced lung injury (104). This suggests that amphiregulin has different functions depending on the cell type and biological background.

### Mechanism underlying the induction of fibrosis by IL-4 and IL-13

Several cell types, including fibroblasts, epithelial cells, and macrophages, are known to induce fibrosis in response to Th2 cytokines (**Figure 4**). Fibroblasts play an important role in normal tissue repair and the induction of fibrosis by directly depositing ECM in response to various growth factors and cytokines, including Th2 cytokines (11). In particular, IL-13 signaling is important in the induction of fibrosis by fibroblasts (8). Pathological fibrosis is caused by direct signaling of IL-13 in PDGFRB^+^ fibroblasts, suggesting that the duration and magnitude of the IL-13 response may determine whether or not tissue repair evolves into fibrosis (8). In addition, several studies have suggested that senescent fibroblasts are resistant to apoptosis and thereby maintain inflammation and fibrosis by producing inflammatory cytokines, immunomodulators, proteases, and growth factors (115, 116). Therefore, senolytic and senotherapeutic drugs may have potential applications in the treatment of fibrosis and aging-related diseases (115, 116). In addition to being an important source of cytokines that trigger type 2 immune responses, epithelial cells—another cell type that induces fibrosis—can directly respond to IL-4 and IL-13 to induce important functions of type 2 immune responses, such as mucus secretion (9). IL-4Rα deficiency in epithelial cells was reported to completely diminish fibrosis during schistosomiasis and bile proliferation, which was associated with experimental liver fibrosis induced by the overexpression of IL-13 (8). Macrophages are also capable of inducing fibrosis in response to Th2 cytokines. Macrophages in tissues are important regulators of tissue fibrosis and play key roles in the initiation, maintenance, and resolution of tissue damage (87, 88, 117). Tissue macrophages are also important producers of chemokines that attract T cells and fibroblasts, which influence the formation of the fibrotic niche (118).

The macrophages that are activated by IL-4 and IL-13 have been reported to induce fibrosis by various mechanisms. First, IL-4 and IL-13 promote fibrosis as well as tissue repair by inducing fibroblast activation and the development of ECM-producing myofibroblasts through the promotion of TGF-β1 production from macrophages (87). Second, MMP12, which is also produced by macrophages, plays a role. MMP12 suppresses the expression of collagen-degrading proteins MMP2, MMP9, and MMP13, which cause an increased fibrotic response and decreased matrix degradation after infection (119). Furthermore, resistin-like alpha (RELMα) secreted by macrophages activates fibroblasts and upregulates lysyl hydroxylase 2, which controls mechanical cross-linking of collagen fibrils (120), resulting in the production of type 2 cytokines (120). In contrast, there was a report showing that macrophages may inhibit fibrosis and inflammation by competing for essential metabolites, such as l-arginine with T cells and other cells. In that report, mice with tissue macrophage-specific deletion of IL-4Rα showed increased inflammation after *Schistosoma mansoni* infection but little change in their liver fibrosis status (91, 121). In other words, the role of macrophages that are activated by IL-4 or IL-13 may vary depending on the organ or pathogenesis (122).

### Diversity of eosinophils involved in fibrosis

Eosinophils play an important role in fibrosis in the lungs and skin in chronic asthma and atopic dermatitis, respectively. In addition, vascular injury is induced by eosinophils after high-dose irradiation through various pro-fibrotic cytokines, including osteopontin, and eosinophil granule proteins (84, 123–125). In contrast, studies using eosinophil-deficient ΔdblGATA and TgPHIL mice demonstrated almost normal fibrosis, granulomatous inflammation, and type 2 cytokine production after infection (126). Thus, the importance of eosinophils in fibrosis remains controversial. It has also been reported that—in Th2 cell-driven allergic inflammation—a subset of eosinophils that are recruited to the tissue enhances inflammation, whereas another subset of eosinophils that is retained in the tissue prior to the induction of inflammation suppresses inflammation *via* the inhibition of bone marrow-derived dendritic cell (BMDC) maturation (127). Therefore, while the recruited eosinophil subset induces an inflammatory response and promotes the development of Th2 cell-dependent fibrosis, it is also possible that the tissue-retaining eosinophil population suppresses fibrosis *via* the suppression of inflammation (127). The existence of such functionally distinct populations of eosinophils may suggest the multi-functional roles of eosinophils in the induction and resolution of fibrosis.

### Mechanism underlying the induction of fibrosis by alarmins

Alarmins released from damaged tissues, such as IL-33, TSLP, and IL-25, have been reported to contribute not only to the induction of Th2 cell differentiation but also the initiation and progression of tissue fibrosis. For example, IL-33 induces the production of IL-13 by ILC2, macrophages (128), and eosinophils (129), as well as the activation of Tpath2 cells (130), as described above. It has been proposed that fibrosis is promoted through the induction of IL-13 production by eosinophils (129). In fact, IL-33 elevation has been reported in dermal tissue in dermatofibrosis (131, 132), lung and intestinal epithelium in patients with fibrotic colitis and pulmonary fibrosis (129, 133–135), and the liver of mice with liver fibrosis (133). TSLP is also detected in human and experimental colonic fibrosis (136), systemic sclerosis (137–139), pulmonary fibrosis (140, 141), and dermatofibrosis (142). IL-25, the third alarmin that promotes fibrosis, activates ILC2s to produce large amounts of the fibrosis-promoting cytokine IL-13 (143). In fact, the intranasal administration of recombinant IL-25 to mice causes airway inflammation and pulmonary fibrosis *via* the production of connective tissue growth factor (CTGF) and TGF-β1 (144). ILC2s that are activated by alarmins also produce large amounts of IL-5 (50). IL-5 recruits and activates eosinophils in bleomycin-induced pulmonary fibrosis (145). Attempts have been made to inhibit tissue fibrosis by inhibiting these alarmins. In mouse models of chronic liver fibrosis and acute pulmonary fibrosis, individual blocking of IL-33, TSLP, and IL-25 had no effect on IL-13-mediated pathology, but simultaneous blocking of these three alarmins significantly reduced fibrosis, eosinophilia, inflammation, and ILC2 mobilization (146). These results suggest that there may be redundancy in the fibrosis-inducing functions of IL-33, TSLP, and IL-25.

### Lung fibrosis in COVID-19

The elucidation of the mechanisms underlying tissue repair and fibrosis by Th2 cells and Th2 cytokines is an urgent issue, as COVID-19, the disease caused by severe acute respiratory syndrome coronavirus-2 (SARS-CoV-2) infection, which is currently causing a global pandemic (147), is accompanied by fibrosis (148–151) and is particularly dangerous for patients with pulmonary fibrotic diseases (152). In patients with severe COVID-19, T helper lymphocytes were found to be biased toward a Th2-like phenotype (153). It has also been reported that miRNAs encoded by SARS-CoV-2 target IL-5 and IL-33 for transcriptional regulation, suggesting that Tpath2 cells may be associated with the pathology of COVID-19 (154). The enhancement of the type 2 immune response was detected in fatal SARS-CoV-2 infection (155) and severe COVID-19 cases (156, 157), and has been reported to be associated with adverse effects in other respiratory infections (158, 159). A meta-analysis of more than 50,000 hospitalized COVID-19 patients showed that the incidence of acute respiratory distress syndrome (ARDS) increased to 14.8% (160). In contrast, it has been observed that the prognosis of COVID-19 is not altered in patients with asthma and other eosinophil-related diseases (161, 162). Similarly, although preclinical studies suggest that eosinophils have potential antiviral activity, there is no evidence that eosinopenia induced by anti-eosinophil therapy increases susceptibility to SARS-CoV-2 (163). In addition, it is noteworthy that no eosinophilia has been observed in the lungs of COVID-19 patients (163). Thus, the inflammatory response induced by conventional or pathogenic Th2 cells and the subsequent fibrosis would be expected to have different effects on the pathology of COVID-19. Pulmonary fibrosis, a common signature in patients with virus-associated respiratory diseases, is also observed in the lungs of patients with SARS-CoV infection (164–166) and Middle East respiratory syndrome coronavirus (MERS-CoV) infection (167). In contrast, it has been reported that pulmonary fibrosis is a risk factor for severe COVID-19 infection (168). An international multicenter study reported that the survival rate of patients who were hospitalized with COVID-19 with complication by non-idiopathic or idiopathic pulmonary fibrosis was significantly lower than that of patients without pulmonary fibrosis (169). This means that patients with interstitial lung disease (ILD), especially fibrotic ILD, face a lower survival rate after COVID-19 infection than patients without ILD (169). In addition, risk factors for COVID-19 and idiopathic pulmonary fibrosis share many similarities, including age, sex, obesity, diabetes, smoking history, and hypertension, and both diseases begin with lung injury (170, 171). In short, a better understanding of the cellular and molecular mechanisms underlying tissue fibrosis will help us to control fibrosis in patients with severe diseases induced by viruses, including COVID-19.

## Control of tissue fibrosis

### Regulation of lung fibrosis by CD103^low^ and CD103^high^ CD4^+^ T_RM_ cells

Th2 T_RM_ cells have been reported to play an important role in type 2 inflammatory responses in local tissues, but their role in tissue fibrosis induced during chronic inflammation has been unclear. Recently, we reported that tissue-resident CD44^hi^CD69^hi^ CD4^+^ T cells showed the higher expression of fibrotic genes in comparison to circulating CD44^lo^ or CD44^hi^CD69^lo^ CD4^+^ T cells in the lungs of mice with repeated exposure to *Aspergillus fumigatus* (13). Furthermore, CD103^lo^ CD4^+^ T_RM_ cells were defined as pathogenic, while CD103^hi^ CD4^+^ T_RM_ cells were defined as immunosuppressive T_reg_ cells, and they had a different epigenetic and transcriptional program in the inflamed lung. CD4^+^CD103^lo^ T_RM_ cells promoted the pathology of allergic inflammation and fibrotic response by increasing Th2 cytokines, such as IL-4, IL-5, and IL-13. In contrast, CD103^hi^ tissue-resident T_reg_ cells expressed Foxp3 and ameliorated the fibrotic response. Thus, tissue-resident CD4^+^ T cell populations with opposing functionality regulate the pathogenesis of fungal-induced chronic inflammatory response and subsequent fibrotic response in the lung (13) (**Figure 4**).

### Mechanisms underlying the suppression of fibrosis by Th subsets other than Th2 cells

While Th2 cells induce tissue fibrosis through type-2 inflammatory responses, Th1 cells inhibit fibrosis through the production of the pro-inflammatory cytokine IFN-γ (**Figure 4**). First, it was reported that IFN-γ suppresses collagen synthesis by fibroblasts and attenuates fibrosis (172). In addition, Wynn et al. previously used IL-12 for the treatment of *S. mansoni* infection in mice and found that IL-12 not only increased the Th1 cytokine expression by inducing Th1 differentiation from naïve CD4^+^ T cells but also suppressed the inflammatory response induced by Th2 cells (173). IFN-γ upregulates the expression of MMPs, such as MMP-2, MMP-7, MMP-9, and MMP-13, by bone marrow-derived cells, resulting in a dramatic improvement in fibrosis (111). This proteolytic activity can alter the remodeling of ECM and help ameliorate fibrosis  (174). In contrast, the induction of fibrosis by Th1 cells and IFN-γ has been reported in liver injury (175) and fibrotic diseases (176, 177). Therefore, the function of Th1 cells and IFN-γ in the suppression of fibrosis remains controversial. Another subset of cells that have been reported to suppress fibrosis are T_reg_ cells. First, it has been reported that the function of T_reg_ cells is reduced in cystic fibrosis patients infected with *Pseudomonas aeruginosa* (178). Second, in hypertensive mice treated with angiotensin II, the adoptive transfer of T_reg_ cells improved cardiac hypertrophy and fibrosis of the heart (179). Furthermore, in Mdr2-deficient mice with sclerosing cholangitis, low-dose IL-2 treatment induced the proliferation of intrahepatic T_reg_ cells and suppressed biliary injury and fibrosis (180). In contrast, there have been several reports on the exacerbation of fibrosis by T_reg_ cells (181. 182). Thus, it is possible that T_reg_ cells may have different functions in different disease models of fibrosis.

### Inhibition of IL-13 signaling by the decoy receptor, IL-13Rα2

IL-13, a major Th2 effector cytokine for the induction of fibrosis, exerts its effects through a receptor complex containing IL-4Rα and IL-13Rα1. There is also a third high-affinity IL-13 receptor, IL-13Rα2, which is an inducible decoy receptor that inhibits the effector function of IL-13 by sequestering the IL-13 from the IL-4Rα/IL-13Rα1 signaling receptor complex (183). Several cytokines that are derived Th1 and Th17 have been reported to increase IL-13Rα2, suggesting a strong reciprocal regulation of the IL-13 function by Th1 and Th17-type immune responses (184). Indeed, combinations of TNF and several cytokines, including IL-4 or IL-17, have been shown to synergistically promote the expression of IL-13Rα2 and inhibit the ability of IL-13 to upregulate downstream targets in mouse and human fibroblasts (184).

### Targeting IL-4 and IL-13 signaling for fibrosis therapy

A number of studies have reported the continuous activation of signaling *via* Th2 cytokines in fibrosis. Among these Th2 cytokines, IL-4 and IL-13 particularly promote fibrosis, and indeed, IL-4 and IL-13 levels are increased in many lung diseases (185–188). Therefore, blockade with antagonists and/or antibodies that target various aspects of the IL-4 and IL-13 signaling pathways in combination or alone is being explored as a therapeutic approach (189). However, the results of these trials in various diseases have been contradictory, and some trials have shown even worse outcomes in treated patients than in controls (190–192). These results suggest the complexity of the mechanism underlying the induction of fibrosis by IL-4 and IL-13 signaling.

In the course of experimental murine schistosomiasis and in a pulmonary granuloma model, blocking IL-13 notably reduces fibrosis but simultaneously increases IFN-γ production, followed by increased TNF production and necrosis of inflamed tissue, exacerbating lung and liver damage. However, blocking both IL-13 and IFN-γ markedly suppresses fibrosis and eliminates the simultaneous type 1 inflammation and subsequent tissue damage that is observed with anti-IL-13 alone  (193). Similarly, blocking IL-4 or IL-13 in HDM-induced allergy models has been shown to induce considerable neutrophilic inflammation by Th17 cells. However, blocking both IL-13 and IL-17A protects mice from neutrophilic and eosinophilic inflammation and eliminates the associated airway hyperresponsiveness and mucus production, indicating that dual or multiple blockade strategies are effective in combating rebound inflammation (193, 194). Interestingly, some clinical trials in which portions of the IL-4 and/or IL-13 signaling pathways are blocked have shown disappointing results (190–192). This may be due to unintended disruption of important beneficial aspects of IL-4 and IL-13 signaling and/or dysregulation of type 1 and Th17-driven inflammatory responses. In contrast, anti-IL-5 therapy in asthmatic patients is reported to inhibit lung fibrosis without affecting the lung function (195). The result of this clinical trial is expected to be attributed to the recruitment and activation of eosinophils by IL-5 (196, 197). Based on these results, it is expected that targeting IL-5 signaling to specifically inhibit the Tpath2 cell function, rather than targeting IL-4 and IL-13 signaling to inhibit the function of whole Th2 cells, will allow for more precise inhibition of tissue fibrosis. Taken together, these studies suggest that careful targeting of therapeutic agents and consideration of dosage is needed to successfully block the pathological features of persistent type 2-driven inflammation. An ideal treatment would neither sacrifice the beneficial functions (e.g., wound repair and epithelial regeneration) nor induce detrimental relapsing inflammatory responses.

## Conclusions

In this review, we provide a systematic overview of the role of conventional Th2 cells in the sequence of tissue inflammation, repair, and fibrosis, and discuss the role of Tpath2 cells (**Table 1**). As described above, Th2 cells play an important role in type-2 inflammation, tissue repair and tissue fibrosis *via* Th2 cytokines, which target various immune and non-immune cells. Among Th2 cells, Tpath2 cells have the ability of higher IL-5 production, which induces eosinophilic inflammation. Another subpopulation of Tpath2 cells, which produces amphiregulin, reprograms recruited-eosinophils to produce osteopontin, the fibrosis-inducing immunomodulatory protein. Furthermore, these Tpath2 cells show the signature of tissue residency. In conclusion, Tpath2 cells are expected to induce subsequent tissue fibrosis in addition to the previously reported induction of inflammatory responses.

**Table 1:** The roles of conventional Th2 cells and Tpath2 cells in inflammation, tissue repair, and fibrosis.

Cell type	Situation	Role	Reference
Conventional Th2 cells	Inflammation	Exacerbation of airway inflammation Exacerbation of atopic dermatitis Exacerbation of eosinophilic esophagitis Exacerbation of inflammation against parasites infection Exacerbation of inflammation against *Aspergillus fumigatus*	(37) (38) (40) (56) (72) (74) (53) (55) (65) (44) (45) (119) (13)
	Tissue repair	Induction of the tissue repair response of macrophage Induction of the hepatocyte proliferation by secreting IL-4 Activation of myofibroblasts by IL-13	(7) (94) (97) (8)
	Fibrosis	Exacerbation of skin fibrosis Exacerbation of lung fibrosis Exacerbation of lung fibrosis by increasing MMP12 *via* IL-13 Exacerbation of skin fibrosis by activating macrophage	(84) (125) (118) (13) (185) (119) (120)
Tpath2 cells	Inflammation	Exacerbation of airway inflammation Exacerbation of atopic dermatitis Exacerbation of eosinophilic esophagitis	(39) (56) (66) (70) (52) (55) (63) (53) (65)
	Fibrosis	Exacerbation of lung fibrosis *via* eosinophil	(12)

Very recently, various novel mechanisms—beyond type-2 inflammation—that induce tissue fibrosis have emerged. For example, it has been reported that altering the gut microbiota may reduce fibrosis (198, 199). Checkpoint inhibitors for cancer treatment have recently received a great deal of attention, but they also show promise in relation to the resolution of fibrosis. It has been reported that checkpoint inhibitors that block costimulatory signals, such as CTLA4 and OX40L, can prevent fibrosis and induce the regression of established fibrosis (200–202). Liver oxidative stress associated with obesity has also been shown to be involved in T cell recruitment and fibrosis (203). Single-cell sequencing technology identifies the transcriptome profiles of individual cells in all cell types in certain tissues, and has allowed us to identify several cell populations that are important for the induction of fibrosis and also novel mechanisms in fibrogenesis (204, 205). In the near the future, this multifaceted approach will hopefully pave the way to the elucidation of the mechanism underlying the induction of fibrosis by Tpath2 cells.

## Author contributions

Writing, peer-review, and editing: KK, AO, MK, KT, and KH, TN. All authors contributed to the article and approved the submitted version.

## Funding

This work was supported by the following grants from the Ministry of Education, Culture, Sports, Science and Technology (MEXT Japan) (JP19H05650, JP18H05375, JP20K20383, JP20H03685, JP21H05120, JP21H05121, JP20KK0351, JP19K16683, JP21K20754, JP22H02885, JP22K15485, JP22K15484); the Japan Agency for Medical Research and Development, AMED (JP21gm1210003, JP21ek0410060, JP21ek0410082, JP22ek0410092); JST FOREST Project (JPMJFR200R); MSD Life Science Foundation; Takeda Science Foundation; and Kowa Life Science Foundation.

## Conflict of interest

The authors declare that the research was conducted in the absence of any commercial or financial relationships that could be construed as a potential conflict of interest.

## Publisher’s note

All claims expressed in this article are solely those of the authors and do not necessarily represent those of their affiliated organizations, or those of the publisher, the editors and the reviewers. Any product that may be evaluated in this article, or claim that may be made by its manufacturer, is not guaranteed or endorsed by the publisher.
